# Visible-Light Active Flexible and Durable Photocatalytic Antibacterial Ethylene-co-vinyl Acetate—Ag/AgCl/α-Fe_2_O_3_ Composite Coating

**DOI:** 10.3390/nano12121984

**Published:** 2022-06-09

**Authors:** Svetlana Vihodceva, Andris Šutka, Maarja Otsus, Heiki Vija, Liga Grase, Anne Kahru, Kaja Kasemets

**Affiliations:** 1 The Institute of Materials and Surface Engineering, Faculty of Materials Science and Applied Chemistry, Riga Technical University, 7 Paula Valdena Str., LV-1048 Riga, Latvia; andris.sutka@rtu.lv (A.Š.); liga.grase@rtu.lv (L.G.); 2 National Institute of Chemical Physics and Biophysics, Laboratory of Environmental Toxicology, Akadeemia tee 23, 12618 Tallinn, Estonia; maarja.otsus@kbfi.ee (M.O.); heiki.vija@kbfi.ee (H.V.); 3 Estonian Academy of Sciences, Kohtu 6, 10130 Tallinn, Estonia

**Keywords:** nanoparticles, antibacterial, photocatalysis, hematite, coating, silver, silver chloride

## Abstract

When particles are mixed in polymer, particle surfaces become passivated by polymer matrix, leading to significantly reduced photocatalytic and, thus, also reduced antibacterial activity, as the catalytic particles become isolated from the outer environment and microorganisms reaching the surface. Herein, we demonstrate a facile and rapid approach for coating preparation at room temperature, yielding good adhesion of particles in combination with the particles’ interface location. Flexible ethylene-co-vinyl acetate Ag/AgCl/α-Fe_2_O_3_ composite coatings were prepared by the spin-coating method. The synthesized photocatalytically active coating surface exhibited a distinct and rapid inhibition of bacterial growth, with at least a 7-log reduction of gram-positive bacteria *Staphylococcus aureus* viability after 30 min of visible-light illumination. We also analyzed the shedding of the Ag-ions and reactive oxygen species production from the composite coating and showed that reactive oxygen species played the main role in the photocatalytic bacterial inactivation, destroying the bacteria cell as proven by the Confocal Laser Scanning Microscopy.

## 1. Introduction

Photocatalytically active antibacterial coatings incorporating metal oxide particles have shown great potential as effective non-targeted disinfectants to eliminate a wide range of microorganisms [1]. Furthermore, photocatalytically active antibacterial coatings, in addition to the elimination of bacteria, also lead to the photooxidation of bacterial debris from the coating surface allowing to prolong antibacterial activity of the coated surfaces [2,3,4]. Thus, photocatalytic surfaces can be applied, for example, on “high-touch surfaces” in hospitals that are frequently touched by the healthcare workers and patients, to avoid the transmission of microorganism [5], but may be also used as flexible films for light-activated rapid sterilization of infected wounds. The major requirement for photocatalytic coatings is that the photocatalytic efficacy must be at its highest level, thus, the photocatalyst must not be completely covered by the other coating components (e.g., polymer), as the photocatalytic particles become isolated from the outer environment and microorganisms reaching the surface [5]. Numerous coating techniques have been used to obtain photocatalytic coatings depending mostly on the application, substrate material, and the shape of the substrate to be coated. Spin-coating is a straightforward method that allows us to obtain reproducible and uniform coatings on relatively flat surfaces, the coating thickness and morphology can be easily adjusted by selecting the appropriate rotation speed, time, and/or depositing several layers [6,7,8].

In this paper, we demonstrate the straightforward, facile, and rapid process of preparation of flexible and durable photocatalytic coating by embedding photocatalytic Ag/AgCl/α-Fe_2_O_3_ composite particles into an ethylene-co-vinyl acetate (EVA) polymer coating top-surface. The polar random units of vinyl acetate groups offer EVA polymer excellent flexibility and fracture toughness [9]. EVA coating with composite could be prepared as a coating to be applied on different substrates, but also as a ‘free-standing’ flexible film or substrate of various thicknesses. The proposed method provides good particle adhesion in combination with the particles’ interface location without the particles being completely covered by the polymer matrix.

Wang et al. (2008) [10] first synthesized Ag/AgCl particles with efficient and stable plasmonic visible-light activated photocatalytic properties, due to the combination of the plasmon resonance of noble-metal nanoparticles with a semiconductor catalyst and efficient electron–hole separation [11,12]. Hematite (α-Fe_2_O_3_), the most stable state of iron oxide due to its relatively narrow band gap of 2.0–2.2 eV, is one of the most extensively investigated materials in photocatalytic degradation of numerous organic contaminants [13,14]. However, there are also some disadvantages such as poor charge transfer properties and fast recombination of charged species, i.e., the high electron–hole recombination efficiency [13,15], but these disadvantages can be amended by synthesis of heterostructures, composites, and/or doping with other materials [13,16]. Due to the advantages listed above, Ag/AgCl can be used to increase the photocatalytic activity of conventional photocatalysts such as α-Fe_2_O_3_, whereas α-Fe_2_O_3_ can enhance the activity and stability of the Ag/AgCl [17]. The AgCl generally could not be excited due to its relatively wide band gap 3.25 eV [18], but in the case of coupling with Ag and α-Fe_2_O_3_ it was observed that the plasmon-induced electrons of the Ag nanoparticles could transfer to the surface of AgCl, while the holes remain on the Ag nanoparticles [19], and electrons in the conduction bands of the AgCl could further transfer to α-Fe_2_O_3_, thus promoting the efficiency of electron–hole separation [20].

Ai et al. (2019) [20] reported that powder γ-Fe_2_O_3_/Ag/AgCl/g-C_3_N_4_ composite particles dispersed in an aqueous environment showed high efficiency in visible-light activated photocatalytic eliminating of gram-negative bacteria *Escherichia coli*, reaching the bacterial removal efficiency log 5.59 after 1 h of illumination, decreasing in the third and fourth cycles to log 3.17 and log 2.32, respectively. Xu et al. (2016) [17] showed that powder Ag/AgCl/@Fe_2_O_3_ (α-Fe_2_O_3_ and y-Fe_2_O_3_) composite particles can fully eliminate *E. coli* (10^4^ CFU/mL) after 30 min of visible-light illumination, however, efficiency gradually decreased during the second and third cycles. The observed decrease in photocatalytic and as a result antibacterial efficiency could be due to the agglomeration of the photocatalytic composite particles [17,19].

In this work, the Ag/AgCl/α-Fe_2_O_3_ composite particles (~2 µm) were synthesized and embedded into EVA (ethylene-co-vinyl acetate) coating top surface, thus flexible antibacterial photocatalytic coatings with composite particles interface location were obtained. The antibacterial efficacy of the prepared photocatalytic EVA-Ag/AgCl/α-Fe_2_O_3_ composite coatings was evaluated against gram-positive bacteria *Staphylococcus aureus*. For that, we used an in-house test protocol with bacterial exposure in the dark (control) and under visible-light illumination. A low-organic growth medium at room temperature was used to mimic the real-life conditions. Obtained coatings showed good durability and high activity against *S. aureus* with at least 7-log reduction during the 3 cycles of use and after 5000 touching times. The mechanism of antibacterial action of the synthesized coatings was analyzed by measuring the production of reactive oxygen species (ROS) and by quantifying the shed Ag-ions from the coatings using the total reflection X-ray spectroscopy (TXRF). The viability of the bacteria was evaluated by live/dead staining with Confocal Laser Scanning Microscopy (CLSM).

## 2. Materials and Methods

### 2.1. Materials

Sodium chloride (NaCl, ≥99%, Taufkirchen, Germany), sodium hydroxide (NaOH, Prague, Czech Republic), silver nitrate (AgNO_3_, >99.8%, Gillingham, UK), 2′, 7′ Dichlorofluorescin diacetate (C_24_H_16_Cl_2_O_7_, ≥98%, Rehovot, Israel), ethylene-co-vinyl acetate polymer (EVA, 40 wt.%, Taufkirchen, Germany), dichloromethane (CH_2_Cl_2_, Taufkirchen, Germany), poly(vinyl pyrrolidone) (PVP, Mw 1,300,000 by LS, St. Louis, MO, USA) and phosphate buffered saline (PBS, pH 7.4, Taufkirchen, Germany) were purchased from Sigma Aldrich and used as received. Iron (III) nitrate nonahydrate (Fe(NO_3_)_3_∙9H_2_O, ≥98%, Darmstadt, Germany), hexane (C₆H₁₄, ≥97.0%, Rosh-Ha’ayin, Israel), and Nutrient agar (NA, Darmstadt, Germany) were purchased from Merck. Tryptone, yeast extract, meat extract, and agar were obtained from LabM (Heywood, UK). All chemicals were purchased as analytical reagent (AR) grade and used without further purification. High purity deionized (DI) water obtained from a Milli-Q^®^ system (Merck Millipore, Darmstadt, Germany) was used for all the tests.

### 2.2. Synthesis of the Ag/AgCl/α-Fe_2_O_3_ Composite

α-Fe_2_O_3_ was prepared by co-precipitation method followed by calcination, as described in our previous works [14,21]. Briefly, 0.1 M iron(III) nitrate solution was prepared, after adding a 0.4 M NaOH solution. Dark brown sediment was formed, then the solution was stirred for 30 min at room temperature and then transferred and heated in a closed 250 mL glass bottle for 72 h at 60 °C. Then, the synthesized FeOOH nanowires were collected and washed by centrifugation (Sigma 2-16P) until neutral pH was obtained, dried at 85 °C overnight, and calcinated at 800 °C for 20 min to obtain α-Fe_2_O_3_ nanowires.

Ag/AgCl/α-Fe_2_O_3_ composite was synthesized by hydrothermal method. Firstly, solution *A* was prepared as reported by Han et al. (Han 2011) [22] with a slight modification: 0.200 g of AgNO_3_ and 0.680 g of PVP were added to 30 mL of DI water and stirred for 1 h at room temperature until the transparent solution was obtained. Then, 0.200 mL of CH_2_Cl_2_ was added and stirred for 30 min at room temperature. Secondly, 0.400 g of as-prepared α-Fe_2_O_3_ nanowire powder was distributed in 30 mL solution *A* by 2 min ultrasonic treatment at room temperature, then the suspension was transferred into a Teflon-lined stainless-steel autoclave (50 mL). The hydrothermal synthesis was conducted at 150 °C for 6 h and then allowed to cool to ambient temperature. After synthesis, composite particles were washed by centrifugation (Sigma 2-16P) until neutral pH was obtained and then dried at 85 °C overnight.

### 2.3. Ethylene-co-vinyl Acetate (EVA) Polymer Coating Preparation

For the preparation of the polymer coating, 5 g of EVA polymer granules were dissolved in 15 mL of hexane by vigorous stirring at 40 °C temperature for 3 h. The resulting suspension was deposited on 1 × 1 cm microscope cover glasses using the spin-coating technique (4000 rpm, 20 s. The resulting surfaces were heated at 40 °C for 2 h.

### 2.4. EVA-Ag/AgCl/α-Fe_2_O_3_ Coating Preparation

For deposition, the synthesized Ag/AgCl/α-Fe_2_O_3_ composite was homogenized in hexane in a concentration of 0.100 g/mL by using an ultrasonic probe at 40 W for 3 min once after preparation (Branson Digital Sonifier^®^, Danbury, CT, USA). 50 µL (0.005 g/cm^2^) of Ag/AgCl/α-Fe_2_O_3_ particle suspension was deposited on EVA-coated cover glasses using the spin-coating technique (4000 rpm, 20 s). The resulting surfaces were heated at 40 °C for 2 h.

### 2.5. Physicochemical Characterization of the EVA-Ag/AgCl/α-Fe_2_O_3_ Composite Coating

The crystalline phase of the synthesized particles was analyzed by Rigaku X-ray diffractometer (XRD, Tokyo, Japan) with Cu Kα radiation in the scan range of 10–90°. The morphology of particles in powder form and as coatings were investigated using the FEI Nova NanoSEM 650 field emission scanning electron microscope (FESEM) coupled with an energy dispersive spectroscopy (EDS) (TEAM™ Integrated EDS with Apollo X SDD) for X-ray microanalysis was used to determine the chemical composition of the samples. Samples were fixed on an aluminum stub with vacuum-resistant carbon tape. FESEM analysis was performed using a low-vacuum mode. An acceleration voltage of 15 kV and a working distance of around 5 mm was typically used.

### 2.6. Evaluation of Antibacterial Efficacy of Photocatalytic EVA-Ag/AgCl/α-Fe_2_O_3_ Composite Coating

The antibacterial efficiency of photocatalytic EVA-Ag/AgCl/α-Fe_2_O_3_ composite coatings was determined against gram-positive bacteria *S. aureus* ATCC 6538 (obtained from the American Type Culture Collection, ATCC) under visible-light (20 W × 2 LED lamp, 4000 lm, set up size 10 × 13.5 cm and intensity 20 Klux, with cooling) in low-organic growth medium at room temperature to mimic the real-life use. Surfaces coated with EVA without photocatalytically active composite particles and with EVA-hematite particles coating were used as negative controls. Suspension of test bacteria at optical density (600 nm) of 0.05, corresponding to 10^7^ CFU/mL, was prepared in 500-fold diluted nutrient broth (NB) (undiluted NB contained 3 g/L meat extract, 10 g/L peptone, 5 g/L sodium chloride in deionized water). The photocatalytic antibacterial test was carried out in a 24-well plate, which contained 500 µL of *S. aureus* suspension in 1:500 diluted NB solution, dipped in bacteria suspension 1 × 1 cm EVA-coated glass, 1 × 1 cm EVA-hematite coated glass as controls, and 1 × 1 cm EVA-composite coated glass (3 replicates in each group). Tests were performed at room temperature (~24 °C) without shaking. Controlled trials in the dark were carried out simultaneously at the same conditions. After 30 min, to retrieve the bacteria from the sample surfaces, the 24-well plates were shaken fast at Microtitration Plate Shaker (Wallac Plate Shake 1296-001, Germany) for 3 min. Afterward, 200 µL of bacterial suspension from all plate wells was transferred to a 96-well plate for preparation of 10-fold serial dilutions in PBS. Cell viability was assessed by plating on agar: 20 µL of 10-fold serial dilutions were pipetted onto solidified nutrient agar plates. The CFU were counted after incubation of the seeded agar plates for 24 h at 30 °C and the results were expressed logarithmically (the bactericidal rate was calculated using formula (1)). The growth of bacteria was checked also after 48 h. All the antibacterial tests were conducted at least two times with 3 replicates in each group. To evaluate the reusability of the coated surfaces, the used samples were washed in 70% ethanol, air-dried, illuminated for 20 min by ultraviolet (UV) light, and dipped again in fresh bacterial suspension for the next testing cycle. The same coated slides were used 3 times.

(1) $$Log_{10}\;reduction=log_{10}\left(\frac{N_{0}}{N}\right)$$

where viable bacteria count of the initial inoculum (*N*_0_), and viable bacteria count of the inoculum retrieved from control surfaces after 30 min in dark and after visible-light illumination (*N*).

### 2.7. Analysis and Quantification of Ionic Ag Release from the Surface

The release of Ag-ions was measured in the same conditions as the antibacterial test (see Section 2.6), but in abiotic conditions, i.e., without the presence of the bacteria. To mimic the release of ionic Ag in 1:500 NB (a testing medium for *S. aureus* assay, see Section 2.6), 1 × 1 cm glass substrates with EVA-Ag/AgCl/α-Fe_2_O_3_ coatings were dipped in 500 µL of the 1:500 NB and incubated in 24-well for 30 min in the dark and under visible-light illumination, as described in Section 2.6. Then, 400 µL of each suspension was collected from each well. The detached non-soluble fraction of Ag particles was removed by the ultra-filtration tube (Microcon Ultracel YM-10, regenerated cellulose 10,000 MWCO), at the centrifugation (Eppendorf Centrifuge 5415 R, Pykkö) speed 10,000 rpm for 30 min. Afterwards, 1% of concentrated nitric acid (HNO_3_) was added. The concentration of the Ag in the supernatant was analyzed by total reflection X-ray fluorescence spectroscopy (TRXF) using Picofox S2 (Bruker AXS Microanalysis GmbH, Karlsruhe, Germany).

### 2.8. Abiotic ROS Measurement

The analysis of the reactive oxygen species (ROS) produced by coating in abiotic conditions was performed using a fluorescent dye 2′,7′-dichlorofluorescein diacetate (DCF-DA) as described in Aruoja et al. [23]. Briefly, 450 µL of DCF-DA (dissolved in ethanol at 1.3 mM) was afresh deacetylated to DCF by adding 1800 mL of 0.01 M NaOH and keeping in the dark, after 30 min the reaction was stopped by adding 9 mL of 25 mM sodium phosphate buffer (pH 7.4) to prepare a 52 μM DCF solution. The solution was placed on ice and kept in the dark until use. After that, 100 μL of 500-times diluted NB from 24-well with dipped in 500-times diluted NB EVA-coating, EVA-hematite coating, EVA-Ag/AgCl/α-Fe_2_O_3_ composite coating non-illuminated, and after illumination with visible-light 30 min, were pipetted to 96-well black microplate wells and then 100 µL of DCF solution was added.

Fluorescence was recorded at excitation and emission wavelengths of 485 and 527 nm, respectively, using the Fluoroskan Ascent FL microplate reader (Thermo Labsystems, Helsinki, Finland). ROS levels were calculated as fold-increase in fluorescence signal compared to the 500-times diluted NB. Results are given as the mean of three experiments ± standard deviation (SD) is reported.

The abiotic ROS level was calculated as follows:

(2) $$F=\frac{F_{t10\left(sample\right)}}{F_{t10\left(control\right)}}$$

where *F*_*t*10_ (sample) is the fluorescence of the solution from dipped coatings with visible-light illumination in 1:500 NB (*t* = 10 min) with incubation with the fluorescent dye; and *F*_*t*10_ (control) is the fluorescence of the sample in the dark (*t* = 10 min) after incubation with the fluorescent dye. Fluorescence is presented in relative fluorescence units (RFU).

### 2.9. Cell Staining and Confocal Laser Scanning Microscopy (CLSM)

For viability staining of *S. aureus*, 400 µL of *S. aureus* cell suspension from each 24-well without coatings, EVA-coating, EVA-hematite coating, and EVA-Ag/AgCl/α-Fe_2_O_3_ composite coating in the dark and after visible-light illumination were collected and centrifuged at 10,000 rpm for 2 min. The cell pellets were co-stained with a stain mixture of 30 µM propidium iodide (PI) and 5 µM Syto9 in DI water. Samples were incubated for 15 min in the dark at room temperature. 4 µL of stained cells were pipetted on the microscopy slide and covered by an 18 × 18 mm coverslip. Images were acquired immediately after staining using Zeiss LSM 800 confocal laser scanning microscope at a magnification of 20x. For Syto 9 and PI signal detection, excitation/emission track settings of 488 nm/450–550 nm and 561 nm/550–700 nm were used, respectively. Images were processed with ZEN software (Carl Zeiss Microscopy, Jena, Germany).

Additional staining was done to EVA-Ag/AgCl/α-Fe_2_O_3_ composite coated surfaces incubated with the *S. aureus* cell suspension in the dark and after visible-light illumination for 30 min. The surfaces were removed from the cell suspension and covered with a stain mixture of 30 µM PI and 5 µM Syto9 in DI water. After 15 min of incubation in the dark at room temperature the excess stain was removed, and the surfaces were covered by a 24 × 50 mm coverslip. Microscopy was done as described above.

## 3. Results and Discussion

### 3.1. Characterization of EVA- Ag/AgCl/α-Fe_2_O_3_ Composite Coatings

**Figure 1:**
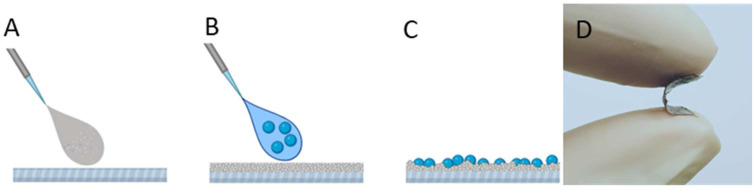
EVA-Ag/AgCl/α-Fe_2_O_3_ composite coating preparation scheme: (**A**) EVA polymer spin-coated onto the glass surface and heated for 2 h at 40 °C, (**B**) composite particles in hexane spin-coated onto the EVA coated surface and heated for 2 h at 40 °C, (**C**,**D**) flexible EVA-coating with interface location of composite particles.

The flexible composite coatings were obtained by spin coating of Ag/AgCl/α-Fe_2_O_3_ composite particle–hexane suspension on EVA film. Since the selected polymer substrate is soluble in hexane by the application of the suspension, the top surface of EVA solubilizes and rapidly solidifies, thus particles from the suspension are strongly adhering. As a result, good adhesion can be obtained in combination with the interface location of exposed particles for strong photocatalytic antibacterial action (scheme, Figure 1).

**Figure 2:**
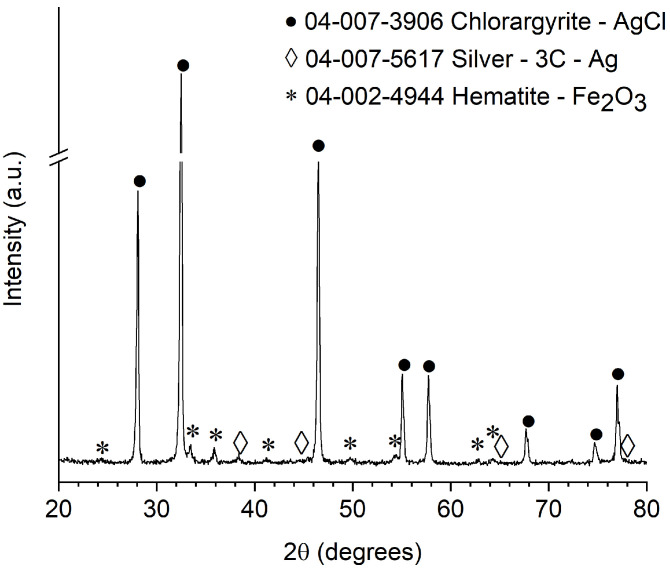
XRD pattern of the Ag/AgCl/α-Fe_2_O_3_ composite. α-Fe_2_O_3_ nanowires XRD pattern can be found in the Supplementary Material (Figure S1).

Figure 2 shows the XRD data of the Ag/AgCl/α-Fe_2_O_3_ composite, XRD pattern confirms that the composite is composed of cubic chlorargyrite AgCl (04-007-3906), metallic Ag (04-003-5617), and rhombohedral hematite structure α-Fe_2_O_3_ (04-002-4944), which indicated that Ag/AgCl/α-Fe_2_O_3_ composite was prepared successfully. The strong and narrow diffraction peaks of the XRD pattern reveal the high crystallinity of composite particles.

**Figure 3:**
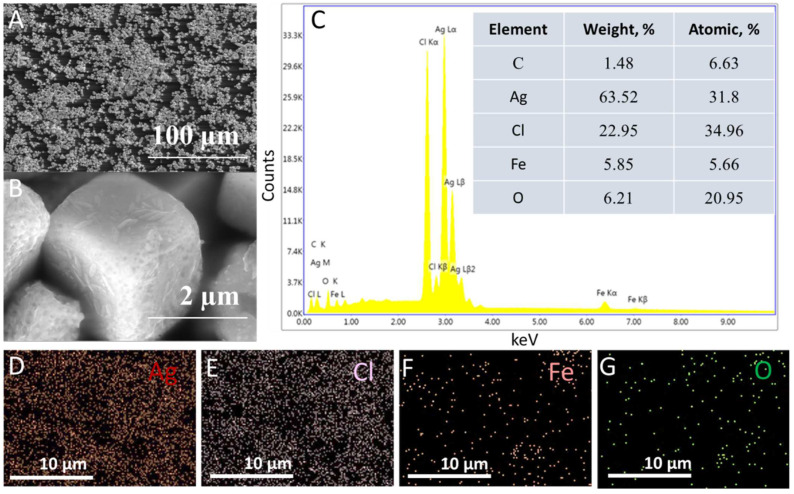
FESEM images low resolution (**A**) and high resolution (**B**) of EVA- Ag/AgCl/α-Fe_2_O_3_ coating. A representative EDS spectrum and table with the atomic and weight percentage of elements (**C**), and elemental mapping of the elements Ag (**D**), Cl (**E**), Fe (**F**), and O (**G**). FESEM images of hematite nanowires can be found in the Supplementary Material (Figure S1).

FESEM-EDS imaging was used to analyze the morphology, microstructures, and elemental content of EVA-Ag/AgCl/α-Fe_2_O_3_ composite coating (Figure 3). A and B images in Figure 3 depict FESEM images of EVA-Ag/AgCl/α-Fe_2_O_3_ composite coating, and the rough surface of the composite is due to the interface location of the composite particles Figure 3A. As shown in Figure 3B, at high magnification the EVA-Ag/AgCl/α-Fe_2_O_3_ composite coating particles have a cube-like morphology with hematite nanowires on the surface. From the FESEM photographs in Figure 3, it is observed that the average size of the composite particles is ~2.2 ± 0.5 µm. An integrated area spectrum is shown in Figure 3C and tabulated with atomic and weight percentages of constituent elements. EDS elemental mapping confirms that coating mainly consists of Ag (Figure 3D), Cl (Figure 3D), Fe (Figure 3D), and O (Figure 3D), and additionally that images show good homogeneity of the coating.

### 3.2. Ionic Ag Release from the Surface in 1:500 Nutriend Broth (NB)

The observed share of shed Ag-ions was slightly higher in the absence of illumination than upon visible-light illumination, 90 ± 10 µg/L, and 72 ± 2.5 µg/L respectively. When AgCl is irradiated by light, the photogenerated electrons could reduce Ag^+^ to Ag^0^ [20,24]. Based on the results, visible-light illumination had no effect on the shedding of Ag-ions from EVA-Ag/AgCl/α-Fe_2_O_3_ composite coating. According to the literature data, these Ag concentrations are not toxic to *S. aureus* [25]. Thus, shedding of the ionic Ag in the observed amount was not toxic to bacteria.

Visible-light Active Flexible and Durable Photocatalytic Antibacterial Ethylene-co-vinyl acetate—Ag/AgCl/α-Fe2O3 Composite Coating.

### 3.3. Photocatalytic Antibacterial Test Results

**Figure 4:**
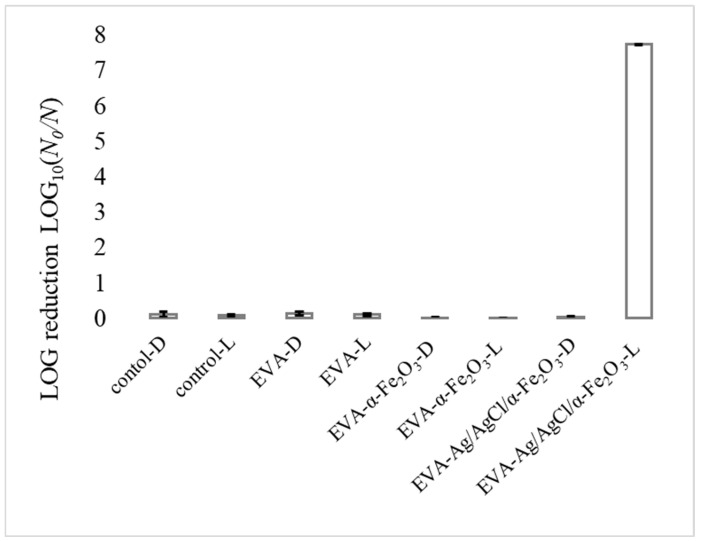
Antibacterial efficiency of EVA-, EVA-α-Fe_2_O_3_, and EVA-Ag/AgCl/α-Fe_2_O_3_ coated surfaces towards *S. aureus* in the dark [D], and upon visible-light illumination [L] for 30 min. Image (B) graph shows log reduction values. The mean value of 2 repetitions with 3 samples in each ± standard deviation (SD) is reported.

As shown in Figure 4, all the *S. aureus* cells were inactivated after 30 min of visible-light illumination using the EVA-Ag/AgCl/α-Fe_2_O_3_ composite coating as a photocatalyst, at least 7-log reduction (full bacteria reduction) of the *S. aureus* cells was achieved, which indicated the high antibacterial performance of the prepared coatings. At the same time, a control experiment revealed that in the presence of visible-light without a photocatalyst coating, as well as in the dark in the presence of EVA-Ag/AgCl/α-Fe_2_O_3_ composite coating there was no reduction of bacterial growth observed. Therefore, the as-prepared photocatalyst composite coating exhibited excellent photocatalytic activity under visible-light irradiation, a high antibacterial activity could be due to the particles’ interface location as a result of high surface area and significant interface area accessible to the outer environment and contact with bacteria. Additionally, visible-light provides additional active sites for contact with bacteria [20,26].

### 3.4. Reusability of The Photocatalytic Coatings

**Figure 5:**
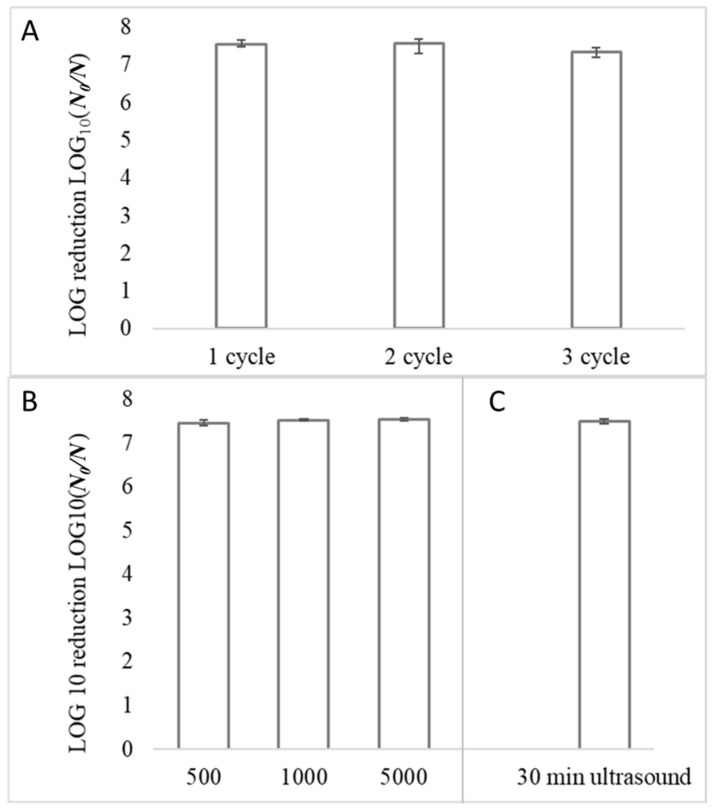
Reusability of EVA-Ag/AgCl/α-Fe_2_O_3_ coating. *S. aureus* > 7-log reduction (full bacteria reduction) was obtained after 30 min of visible-light illumination in all 3 cycles (**A**). Additionally, *S. aureus* > 7-log reduction (full bacteria reduction) remained unchanged after coatings touching 500, 1000, and 5000 times (**B**), and 30 min ultrasound washing (**C**) after 30 min of visible-light illumination. The mean value of 2 repetitions with 3 samples in each (cycles) and 3 repetitions ± standard deviation (SD) is reported.

Since good performance should also include resistance and reuse of the coating, it is necessary to evaluate the reusability as an important characteristic for the practical application of the photocatalytic antibacterial coatings by assaying the same coatings by cycle photodegradation experiments. Therefore, the recovery experiment was conducted by visible-light illumination of the coating for 3 cycles. After each application, coatings were washed with 70% ethanol and rinsed with DI water with afterward illumination with UV light for 20 min. As shown in Figure 5, all the *S. aureus* cells were inactivated after 30 min of visible-light illumination during 3 cycles using the EVA-Ag/AgCl/α-Fe_2_O_3_ composite coating. Furthermore, the decrease in the antibacterial efficiency was not observed even in the third cycle. Specifically, at least a 7-log reduction of the *S. aureus* cells was achieved in all cycles Figure 5A, which indicated the high antibacterial performance of the prepared coatings. These data specified that produced EVA-Ag/AgCl/α-Fe_2_O_3_ coating is stable even after undergoing several catalytic reaction cycles and washings. The coating’s high photocatalytic antibacterial activity and durability during the repeated cycles could be due to the stability of the composite particles as a result of Ag/AgCl α-Fe_2_O_3_ and the prevention of the particle’s aggregation by the incorporation of the composite particles in the EVA polymer surface and its interface location thus uninterrupted contact with outer environment and bacteria along with excellent particles adhesion.

To evaluate how extensive use (touching) affects the photocatalytic antibacterial properties, samples were physically touched with rubber-gloved hands (finger) 500, 1000, and 5000 times. Coatings maintained their antibacterial activity against *S. aureus* even after 5000 contacts. Specifically, the >7-log reduction of the *S. aureus* cells remained unchanged even after 5000 contacting Figure 5B. Additionally, ultrasound washing for 30 min has no impact on coating activity Figure 5C, which indicated the high durability of the prepared coatings through the proposed approach.

### 3.5. Abiotic ROS Measurement

**Figure 6:**
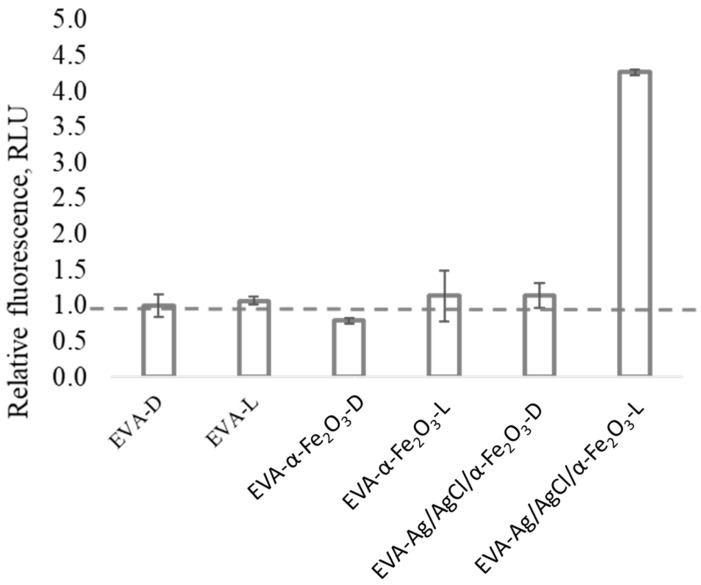
Generation of reactive oxygen species (ROS) measured with fluorescent dye DCF-DA in abiotic conditions (i.e., without the bacterial cells). Relative fluorescence units (RFUs) due to ROS production in the liquid in contact with EVA-coating, EVA-hematite coating, and EVA-Ag/AgCl/α-Fe_2_O_3_ composite coating in the dark [D], and upon visible-light illumination [L].

An increase in reactive oxygen species (ROS) production was observed after EVA-Ag/AgCl/α-Fe_2_O_3_ composite coating illumination with visible light (Figure 6), supporting general radical generation at the photocatalyst–water interface. Therefore, ROS formation by the photocatalytic coatings showed the same trend as that of the antibacterial activity tests, resulting in the photocatalytic coating upon visible-light illumination exhibiting both the highest fluorescence intensity and bactericidal effect. Thereby, the ROS production is likely the main reason for the coating’s activity under visible-light illumination that causes the death of bacteria and destruction of the bacterial cells also proven by CLSM analysis. Ai et al. (2019) found that using γ-Fe_2_O_3_/Ag/AgCl/g-C_3_N_4_, the major reactive oxygen species generated by the photocatalysis are H_2_O_2_ and ·O, this ROS could attack the bacteria cell wall, oxidize and tear the membrane, thus changing their permeability, and afterward react with cellular proteins, causing the leakage of cell ‘inclusions’ and protein denaturation, resulting in the death of the bacteria.

### 3.6. Confocal Laser Scanning Microscopy (CLSM) Results

**Figure 7:**
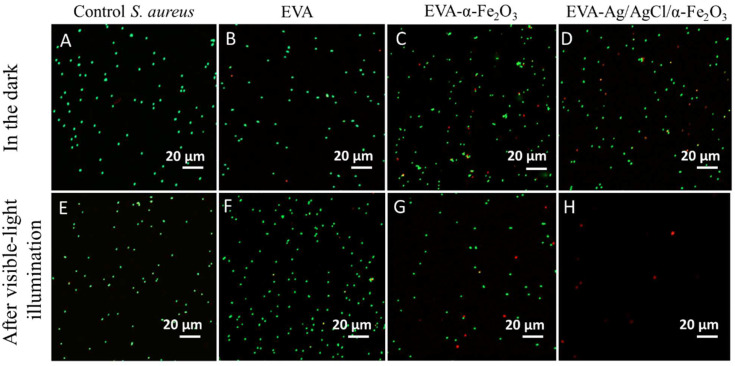
Representative Confocal Laser Scanning Microscopy (CLSM) images of *Staphylococcus aureus* suspension after 30 min exposure to the different surfaces in the dark (**A**–**D**) and under the visible-light illumination (**E**–**H**): viable cells—green (Syto9); dead or membrane damaged cells—red (PI). Scale bars represent 20 µmImages of bacteria growth test in tubes can be found in the Supplementary Material (Figure S2).

Live/dead staining with S9 and PI and CLSM visualization revealed that the EVA-Ag/AgCl/α-Fe_2_O_3_ composite coating under visible-light illumination (but not in the dark) remarkably decreased the number of cells in suspension compared to the bacterial suspension without coating (control), or EVA and EVA-hematite coating surfaces (Figure 7). Red fluorescence of the cells indicates membrane-damaged and non-viable bacteria (PI-positive cells). It can be concluded that the ROS production by the EVA-Ag/AgCl/α-Fe_2_O_3_ coating during the visible-light illumination caused *S. aureus* cell death by disrupting the integrity of the bacterial membrane (Figure 7H).

**Figure 8:**
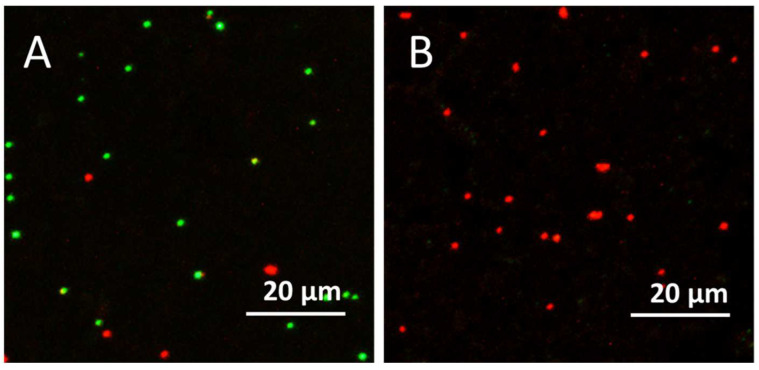
Representative Confocal Laser Scanning Microscopy (CLSM) images of live/dead staining of *S. aureus* on the coating surfaces of EVA-composite in the dark (**A**) and after the visible-light illumination (**B**) for 30 min. Viable cells—green (Syto9); dead or membrane damaged cells—red (PI). Scale bars represent 20 µm.

Live/dead staining and CLSM visualization were also performed for the coating surface of EVA-composite in the dark (Figure 8A) and after the visible-light illumination (Figure 8B). Results revealed that in the dark (Figure 8A) both red (PI-positive) and green (Syto9) cells were determined, but after visible-light illumination, only PI-positive was observed on the surface of the photocatalytic coating, indicating the presence of only dead or membrane-damaged bacterial cells.

## 4. Conclusions

With a flexible and durable photocatalytic antibacterial surface, a visible-light-driven EVA-Ag/AgCl/α-Fe_2_O_3_ composite coating was successfully fabricated by a simple spin-coating method. The prepared EVA-Ag/AgCl/α-Fe_2_O_3_ composite coating showed high efficiency in photocatalytic inactivating of *S. aureus* under visible-light illumination because of the composite particles’ interface location that allows the catalytic particles to contact with microorganisms at the surface. The obtained results showed that after visible-light illumination for 30 min, the average disinfection efficiency reached >7-log reduction of *S. aureus*, even after 3 cycles of use. Moreover, the prepared coatings showed high photocatalytic antibacterial performance in combination with good durability for several photocatalytic and washing cycles. The bactericidal activity of the coating under visible-light illumination was explained by the ROS production.

## Data Availability

The data presented in this study are available on request from the corresponding author.

## Supplementary Materials

The following supporting information can be downloaded at: https://www.mdpi.com/article/10.3390/nano12121984/s1, Figure S1: FESEM images EVA-hematite coating (A), α-Fe_2_O_3_ nanowires at high resolution (B) and XRD pattern of the α-Fe2O3 nanowires (C); Figure S2: Visualization of the tubes and OD density after 8 h incubation at 30 °C temperature.
