# Supplementary material for: Visible-Light Active Flexible and Durable Photocatalytic Antibacterial Ethylene-co-vinyl Acetate—Ag/AgCl/α-Fe2O3 Composite Coating
Source: Nanomaterials (Basel). 2022 Jun 9;12(12):1984. doi: 10.3390/nano12121984 (PMC9227942; doi:10.3390/nano12121984)
Supplement: Supplementary file 1 [file nanomaterials-12-01984-s001.zip › nanomaterials-1700118-supplementary.pdf]

Supplementary Materials

Visible-Light Active Flexible and Durable Photocatalytic Antibacterial Ethylene-co-vinyl acetate—Ag/AgCl/α-Fe_2O_3 Composite Coating

Svetlana Vihodceva [1,*], Andris Šutka [1], Maarja Otsus [2], Heiki Vija [2], Liga Grase [1], Anne Kahru [2,3,*] and Kaja Kasemets [2,*]

[1] The Institute of Materials and Surface Engineering, Faculty of Materials Science and Applied Chemistry, Riga Technical University, 7 Paula Valdena Str., LV-1048 Riga, Latvia; andris.sutka@rtu.lv (A.Š.); liga.grase@rtu.lv (L.G.)

[2] National Institute of Chemical Physics and Biophysics, Laboratory of Environmental Toxicology, Akadeemia tee 23, 12618 Tallinn, Estonia; maarja.otsus@kbfi.ee (M.O.); heiki.vija@kbfi.ee (H.V.)

[3] Estonian Academy of Sciences, Kohtu 6, 10130 Tallinn, Estonia

* Correspondence: svetlana.vihodceva@rtu.lv (S.V.); anne.kahru@kbfi.ee (A.K.); kaja.kasemets@kbfi.ee (K.K.)

1.1. pH of the Ag/AgCl/α-Fe2O3 composite in 1:500 NB.

Ag/AgCl/α-Fe_2O_3 composite in 1:500 NB solution 4 g/L - pH 6.27.

1.2. FESEM images and XRD pattern of EVA-hematite coating and hematite particles.

FESEM images show that synthesized hematite particles have nanowire shape (Figure S1 A-B). The XRD studies indicate that the hematite phase was successfully obtained by so-precipitation synthesis and annealing at 800 °C (Figure S1 C).

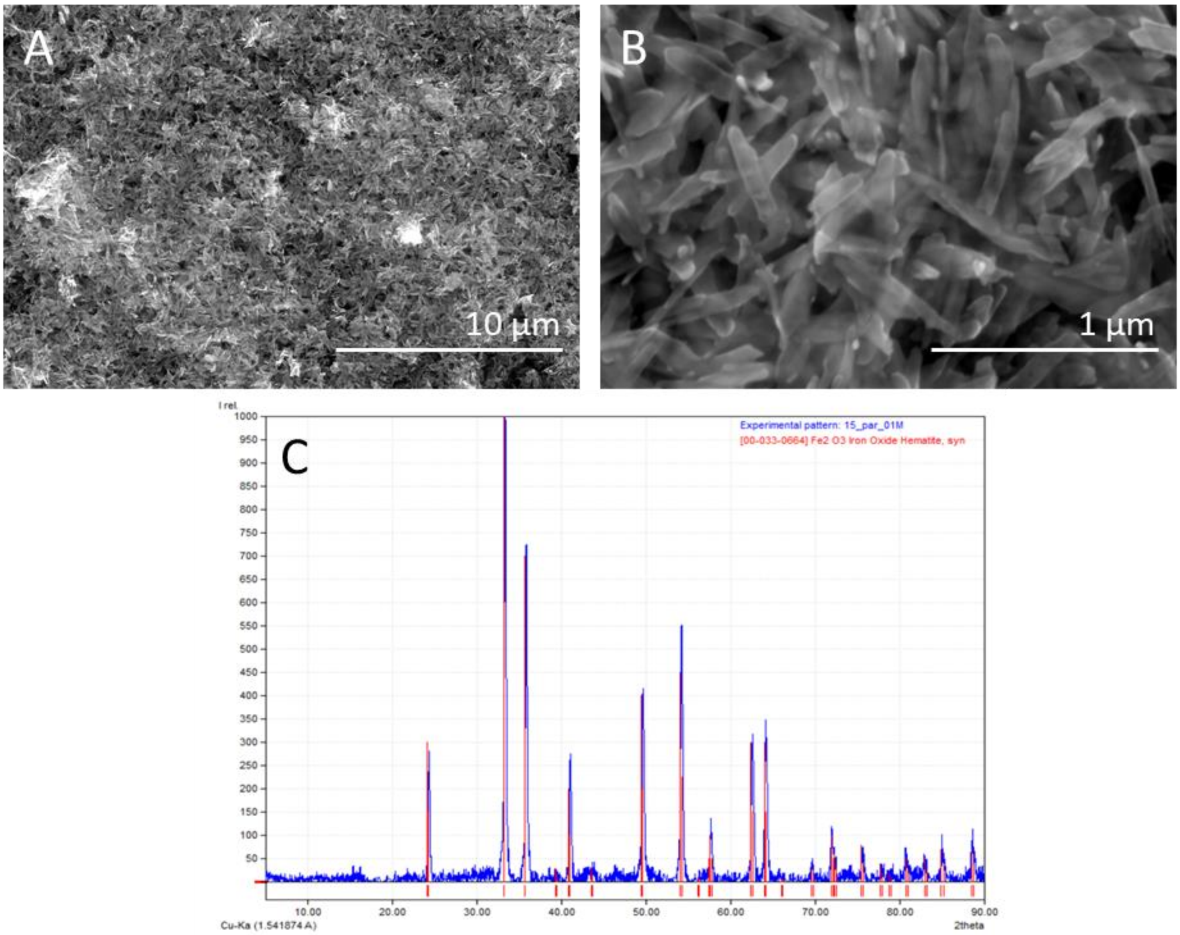

Figure S1. FESEM images EVA-hematite coating (**A**), α-Fe_2O_3 nanowires at high resolution (**B**) and XRD pattern of the α-Fe_2O_3 nanowires (**C**).

1.3. Bacteria in Suspension after Photocatalytic Tests.

To evaluate the presence of bacteria in suspensions after the photocatalytic test 200 µl of suspension from 24-well with EVA and EVA-Ag/AgCl/α-Fe_2O_3 coated surfaces in the absence of illumination [D], and upon visible-light illumination [L] (Figure S2) was transferred to tube with 3 ml of undiluted Nutrient Broth, afterwards bacteria were grown on rotating shaker for 8 h at 30 °C. After 8 h tubes were evaluated visually and OD (600 nm) were measured. Results show that tube with suspension from EVA-Ag/AgCl/α-Fe_2O_3-L - upon visible-light illumination remain transparent with unchanged OD.

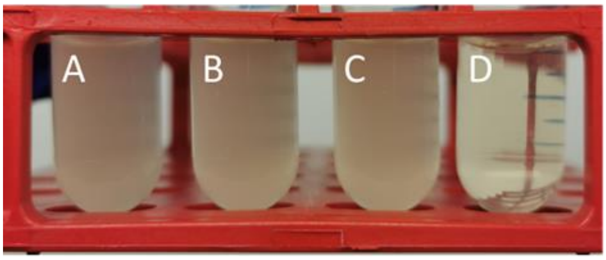

	Sample name	OD_{600}
A	EVA-D	2.370
B	EVA-L	1.810
C	EVA-Ag/AgCl/α-Fe_2O_3-D	2.060
D	EVA-Ag/AgCl/α-Fe_2O_3-L	0.001

Figure S2. Visualization of the tubes and OD density after 8 h incubation at 30 °C temperature.
